# Visualization of argininosuccinate synthetase by *in silico* analysis: novel insights into citrullinemia type I disorders

**DOI:** 10.3389/fmolb.2024.1482773

**Published:** 2024-11-22

**Authors:** Xia Gu, Wenhui Mo, Guiying Zhuang, Congcong Shi, Tao Wei, Jinze Zhang, Chiaowen Tu, Yao Cai, Biwen Liao, Hu Hao

**Affiliations:** ^1^ Department of Neonatology, The Sixth Affiliated Hospital, Sun-Yat-Sen University, Guangzhou, China; ^2^ Biomedical Innovation Center, The Sixth Affiliated Hospital, Sun Yat-sen University, Guangzhou, China; ^3^ Department of Neonatology, Foshan Fosun Chancheng Hospital, Foshan, China; ^4^ Department of Neonatology, The Maternal and Child Health Care Hospital of Huadu, Guangzhou, China; ^5^ Inborn Errors of Metabolism Laboratory, The Sixth Affiliated Hospital, Sun-Yat-Sen University, Guangzhou, China; ^6^ Guangdong Shaohe Biotechnology Co., LTD., Guangzhou, China

**Keywords:** citrullinemia type I, argininosuccinate synthetase, deletion-insertion variants, *in silico* analysis, molecular dynamics simulation

## Abstract

**Background:**

Citrullinemia type I disorders (CTLN1) is a genetic metabolic disease caused by argininosuccinate synthetase (ASS1) gene mutation. To date, the human genome mutation database has documented over 100 variants of the ASS1 gene. This study reported a novel deletion-insertion variant of ASS1 gene and employed various prediction tools to determine its pathogenicity.

**Methods:**

We reported a case of early-onset CTLN1. Whole exome sequencing was conducted to identify genetic mutations. We employed various structure prediction tools to generate accurate 3D models and utilized computational biology tools to elucidate the disparities between the wild-type and mutant proteins.

**Results:**

The patient was characterized by severe clinical manifestations, including poor responsiveness, lethargy, convulsions, and cardiac arrest. Notably, the patient exhibited significantly elevated blood ammonia levels (655 μmol/L; normal reference: 10–30 μmol/L) and increased citrulline concentrations (936 μmol/L; normal reference: 5–25 μmol/L). Whole exome sequencing revealed a in-frame deletion-insertion mutation *c.1128_1134delinsG* in the ASS1 gene of unknown significance, which has not been previously reported. Our finding indicated that the C- terminal helix domain of the mutant protein structure, which was an important structure for ASS1 protein to form protein tetramers, was indeed more unstable than that of the wild-type protein structure.

**Conclusion:**

Through conducting an in silico analysis on this unique in-frame deletion-insertion variant of ASS1, our aim was to enhance understanding regarding its structure-function relationship as well as unraveling the molecular mechanism underlying CTLN1.

## 1 Introduction

Citrullinemia type I (CTLN1) is an autosomal recessive genetic disorder and is the third most common urea cycle disorder (UCD), with an incidence of 1 in 250,000 (Posset et al., 2019; Summar et al., 2013). Argininosuccinate synthetase (ASS1) is a cytosolic enzyme that catalyzes the synthesis of argininosuccinic acid from citrulline and aspartic acid within the urea cycle, primarily expressed in periportal hepatocytes (Ratner, 1973). A deficiency in ASS1 disrupts the urea cycle, leading to hyperammonemia and elevated citrulline levels in the blood, urine, and cerebrospinal fluid. CTLN1 is caused by mutations in the ASS1 gene (Beaudet et al., 1986; Haberle et al., 2019).

The main clinical manifestations of CTLN1 include citrullinemia and hyperammonemia. Depending on the severity of ASS1 impairment, CTLN1 can be categorized into classic (early-onset), mild (late-onset), and asymptomatic (carriers) forms (Haberle et al., 2003; Husson et al., 2003). Children typically present with the disease during the neonatal period, exhibiting symptoms such as gradually worsening disturbances of consciousness, feeding difficulties, and convulsions, which can ultimately lead to cerebral softening, atrophy, poor prognosis, and high mortality (Quinonez and Lee, 1993). However, many clinicians have limited awareness of this condition, resulting in frequent misdiagnoses and missed cases. Biochemical indicators such as hyperammonemia, elevated plasma citrulline, low plasma arginine, and increased urinary orotic acid strongly suggest ASS1 deficiency (Haberle et al., 2019; Woo et al., 2014). Nonetheless, DNA sequencing remains the most definitive diagnostic tool, making it essential to understand the genetic characteristics and pathogenic mechanisms of the ASS1 gene.

The ASS1 gene, located at 9p.34.11, has an open reading frame of 1,239 bp and consists of 16 exons, with the translation start codon in exon 3 and the termination codon in exon 16 (Freytag et al., 1984; Häberle et al., 2002). Variants of the ASS1 gene are heterogeneous; to date, the human genome mutation database has recorded over 100 variants, primarily consisting of missense mutations, while deletion mutations are the least common (Diez-Fernandez et al., 2017). Additionally, the relationship between genotype and phenotype is not yet fully understood (Kimani et al., 2015). Bioinformatics approaches, such as homology modeling and 3D visualization of the target gene sequence, offer promising methods for analysis (Kelley and Sternberg, 2009). Overall, conducting comprehensive functional analyses to study how mutations affect protein function is crucial for understanding the molecular pathogenesis of CTLN1 and interpreting its clinical significance.

In this study, we reported a case of early-onset CTLN1 and detailed a series of detection methods used to analyze the structure-function relationship of an unreported in-frame deletion-insertion mutation, *c.1128_1134delinsG*. Through *in silico* analysis, we elucidated the impact of this novel deletion-insertion variant on protein function, examining its effects on the 3D structure. Computational comparisons of the wild-type and variant proteins revealed the detrimental nature of this mutation.

## 2 Materials and methods

### 2.1 Clinical manifestation

The proband was born at 37 weeks gestation via vaginal delivery, weighing 2,360 g. There was no significant family history of inherited metabolic diseases, and the mother had regular antenatal check-ups. He was diagnosed with neonatal sepsis, shock, and respiratory failure, leading to hospitalization on day four. Blood tests revealed stress-induced hyperglycemia, metabolic acidosis, hyperlactatemia, and slightly elevated blood ammonia levels (123 μmol/L; normal reference: 10–30 μmol/L). After cardiopulmonary resuscitation and treatment for infection and shock, circulatory indices improved, but blood ammonia rose significantly to 655 μmol/L. Tandem mass spectrometry (MS/MS) results indicated elevated citrulline levels (936 μmol/L; normal reference: 5–25 μmol/L), along with increased citrulline/arginine and citrulline/phenylalanine ratios.

Following symptomatic treatment, including protein intake restriction, L-carnitine administration, and ammonia reduction, the patient’s consciousness was restored and muscle tone normalized. However, during follow-up, blood ammonia levels remained slightly elevated, and the patient exhibited speech delay and motor retardation by 6 months of age.

### 2.2 Genetic analysis

For further diagnosis and genetic counseling, 2 mL of peripheral venous blood was extracted from the child and his parents using an EDTA anticoagulant tube after approval by the Ethics Committee and informed consent of the parents. Whole exome sequencing was performed by high throughput sequencing technology (second generation sequencing) in Guangzhou JinYu Medical Laboratory Center. Genomic DNA was extracted with the QIAamp DNA extraction kit (QIAGEN). The Novaseq 6,000 sequencer (Illumina, US) was used in high throughput mode for double-ended 150 bp (Pair End 150 bp) PE150 sequencing.

### 2.3 *In silico* analysis of ASS1 gene

#### 2.3.1 Protein modeling and analysis

The protein sequence of ASS1 (accession number P00966) was harvested from UniProt (https://www.uniprot.org/uniprotkb/P00966).SWISS - MODEL (Waterhouse et al., 2018) and trRosetta (Du Z et al., 2021) were utilized to obtain relatively accurate wild-type and mutant-type protein structures, and the obtained structures were evaluated using SAVES online server (https://saves.mbi.ucla.edu). To verify the reliability of the protein structures obtained through SWISS-MODEL and trRosetta, we used the Procheck tool from SAVES (Structural Analysis and Verification Server) to assess model quality. This method analyzed the backbone dihedral angles of the protein (Ramachandran plot) to evaluate the geometric validity of the model; the larger the area of the most favorable regions, the better the model. Additionally, we selected the ASS1 structure from the Protein Data Bank (Rose et al., 2011) as the reference structure (2nz2.pdb, 412 amino acids) (Karlberg et al., 2008).

#### 2.3.2 Protein conservation analysis

Protein conservation analysis evaluated whether certain sites in a protein’s amino acid sequence had been strictly preserved during evolution by comparing sequences across multiple species. Conserved amino acid sites were often crucial for the protein’s structure and function, as changes in these sites could disrupt normal protein function. WebLogo (Crooks et al., 2004) was used to visualize the degree of amino acid conservation, and CASTp software (Tian et al., 2018)was used to predict the difference in protein structure surface topography before and after mutation.

#### 2.3.3 Molecular dynamics simulation

To further analyzed the dynamic behavior and structural stability of wild-type and mutant ASS1 proteins, we used GROMACS (2024.01) (Abraham et al., 2015) (GROningen MAchine for Chemical Simulations) for molecular dynamics simulations. GROMACS was an efficient molecular dynamics simulation software specifically designed for simulating the behavior of biomolecules. Used the three-dimensional structure generated by trRosetta as the starting conformation, selected the amber14 s b_OL15.ff (Maier et al., 2015) force field suitable for protein molecules. Water molecules were used as solvent (Tip3p water model), and the total charge of the simulation system was neutralized by adding an appropriate number of Na^+^ ions. The simulation system carried out the isothermal isovolumic ensemble (NVT, 309.15K) equilibrium and isothermal isobaric ensemble (NPT, 1 bar) equilibrium for 500,000 steps respectively, and finally conducted a 100 ns production simulation with a time step of 2 fs, recording trajectory data every 10 ps.

We extracted and analyzed the following data from the molecular dynamics simulation: 1) RMSD (Root Mean Square Deviation): compared the changes in the protein relative to the initial conformation during the simulation; 2) RMSF (Root Mean Square Fluctuation): analyzed the fluctuation of each residue to assess the dynamic changes in the mutated regions; 3)Rg (Radius of gyration): The RMS distance between the protein atoms and its axis of rotation. A low Rg value indicates that the protein is more compact and less flexible, while a high Rg value suggests that the protein is less compact and more flexible; 4)SASA (solvent-accessible surface area): The SASA parameter was used to determine the protein surface area accessible to solvent. Protein conformations with high SASA values were more stable; 5) Hydrogen bond analysis: calculated the number of hydrogen bonds within the protein and between the protein and water molecules to evaluate the stability of the structure; 6) PCA(Principal Component Analysis) graph: indicated the diversity of protein conformations or the stability of the system; 7) The contact map analysis; 8) FES (Free Energy Surface): The energy state of a protein under specific conditions, reflecting its stability and variability. Besides, the protein structure was visualized and color-coded using PyMol (Lill and Danielson, 2011) and Chimera (Pettersen et al., 2004).

## 3 Results

### 3.1 Gene test results

**FIGURE 1:**
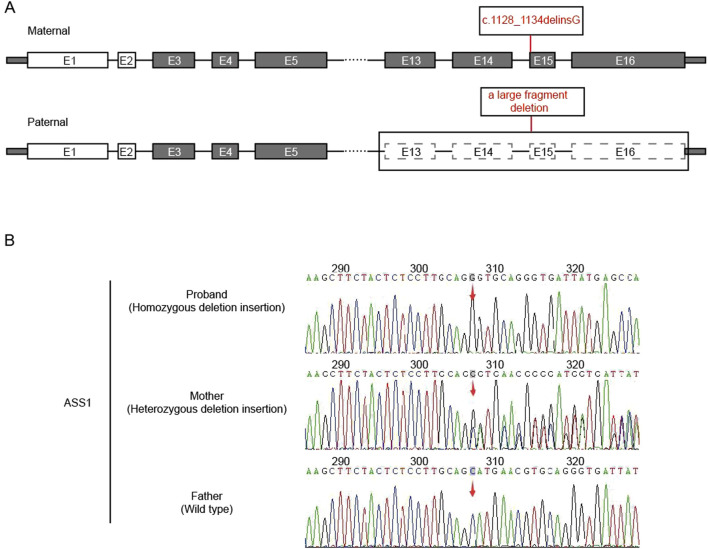
Analysis of ASS1 gene. **(A)** The location of the mutation in the gene structure (E:exons); **(B)** Sanger sequencing result (red arrow: the starting positions of each mutation).

After the sequencing data were filtered by HGMD, OMIM, and Clinvar databases, it was found that there was a deletion-insertion mutation *c.1128_1134delinsG(p.Ser376_Asn378delinsArg)* in the ASS1 gene (NM_000050.4 Exon15) on chromosome 9:133374892, and a large fragment deletion on chromosome 9:133364710-133376418 (11.7 kb, Exon13-16), which were suggested as homozygous mutation by first-generation sanger sequencing (Figure 1). This maternal mutation would cause deletion of amino acids from Ser 376 to Asn 378, which would be replaced by the inserted amino acid Arg, but would not cause frame shift. No literature report was found in HGMD database. ESP6500siv2_ALL, Thousand Genome (1,000 g 2015aug_ALL) and dbSNP147 were not recorded, either. Overall, the clinical significance was not clear. The paternal large fragment deletion was a heterozygous mutation from exon 13–16 of paternal ASS1 gene, which was pathogenic. The data presented in the study are deposited in the GSA-human repository (https://ngdc.cncb.ac.cn/gsa-human/), accession number HRA005194. The human genetic information data involved in this study have been registered in Human Genetic Resource Administration of China (registration number: 2023BAT1206).

### 3.2 ASS1 protein model construction

**FIGURE 2:**
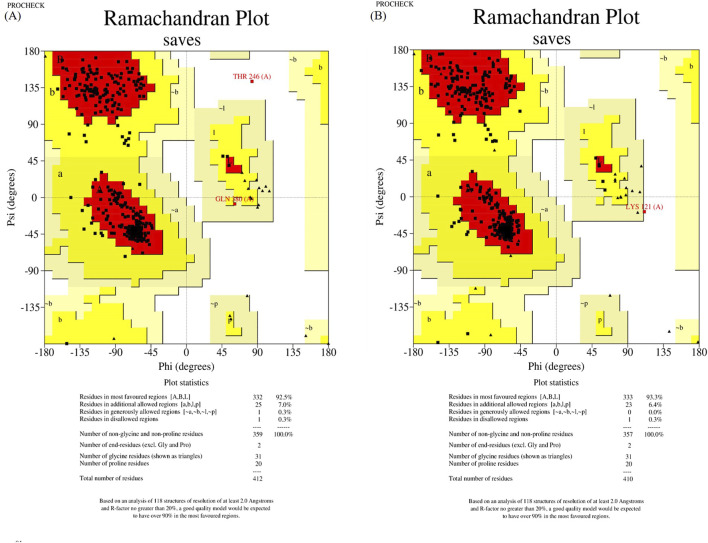
Structure assessment of ASS1 protein structures predicted by trRosetta. **(A)** shows the wild-type ASS1 protein structure evaluation, and **(B)** shows the structural evaluation of the mutant-type ASS1 protein.

The wild-type and mutant structures, constructed by SWISS-MODEL and trRosetta, were evaluated using Ramachandran plot of PROCHECK SAVES v6.0. For further analysis, we designated the structures with the highest scores in the most favored regions as the wild-type and mutant structures, respectively. In a result, wild-type and mutant structures constructed by trRosetta were selected (shown in Figure 2).

### 3.3 Protein conservation analysis and conformation analysis

**FIGURE 3:**
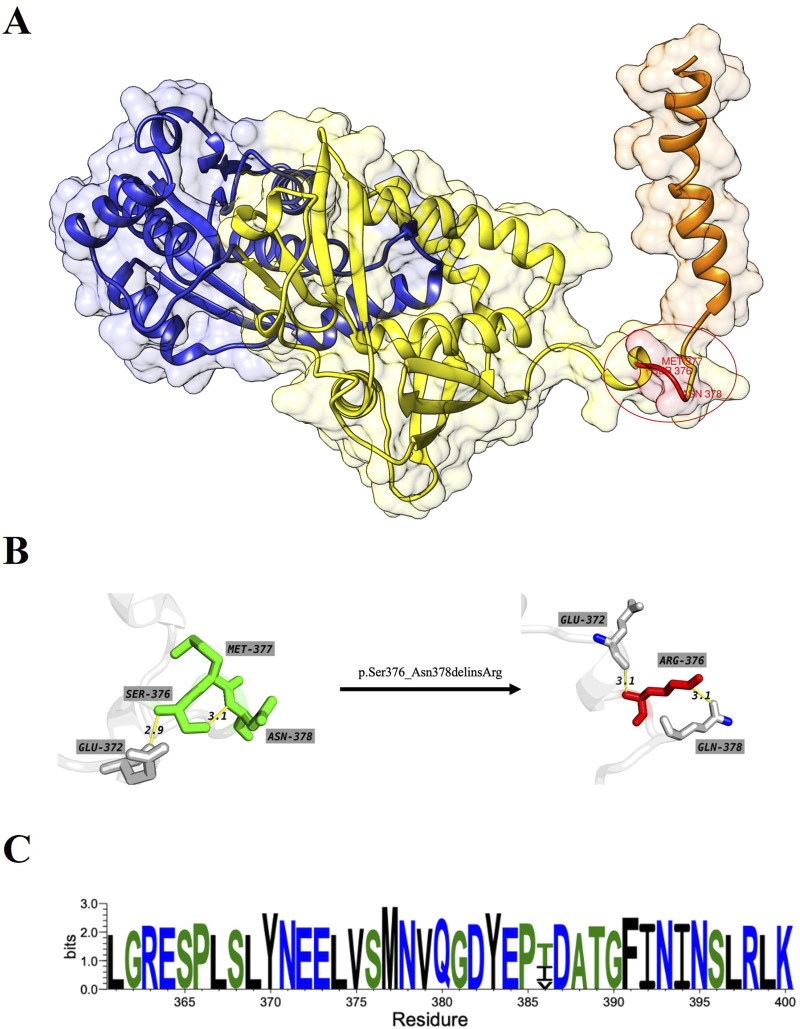
**(A)** represents the wild-type structure of ASS1 protein, in which the nucleotide binding domain is marked in blue, the synthase domain is marked in yellow, the C-terminal helix is marked in orange, and the mutant residue is marked in red. **(B)** represents the interaction between residues before and after mutation, which residues od wild-type structuremaeked in green and those of mutant protein structure marked in red. **(C)** Visualization for the degree of conservation of ASS1 protein sequence.

The overall structure of ASS1 protein consisted of three domains, a nucleotide-binding domain, a synthetase domain and a C-terminal helix involved in oligomerization (shown in Figure 3A). The two first domains were highly integrated, with two connected helices from the nucleotide-binding domain running through the core of the synthetase domain. There was a small loop region between the synthetase domain and the C-terminal helix, and this loop region was extremely flexible, while the mutation site of this case happened to be located in this loop region, and the original SER376, MET377 and ASN378 were deleted, and ARG376 was inserted (shown in Figures 3A, B). The CASTp server results showed that both the surface area and volume of the loop region decreased significantly after mutation (Table 1), which may indicated that the flexibility of the loop region decreased after mutation, and this change was highly likely to have an impact on the rear C-terminal helix, resulting in the inability of the C-terminal helix to maintain its function. WebLogo was used to analyze the conserved degree of 360–400 amino acid of ASS1 protein (shown in Figure 3C). It could be seen that sites 376–378 were highly conserved. These results indicated that the mutation was likely to have an impact on protein structure and function.

**TABLE 1:** Differences of the surface topography between wild-type and mutation by using CASTp server.

	Different in surface topography	Area	Volume
wild-type	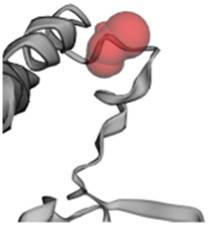	53.668	25.349
mutation	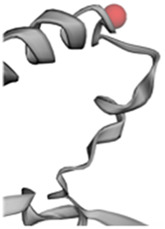	0.805	0.562

### 3.4 Molecular dynamics simulation and structure stability analysis

**FIGURE 4:**
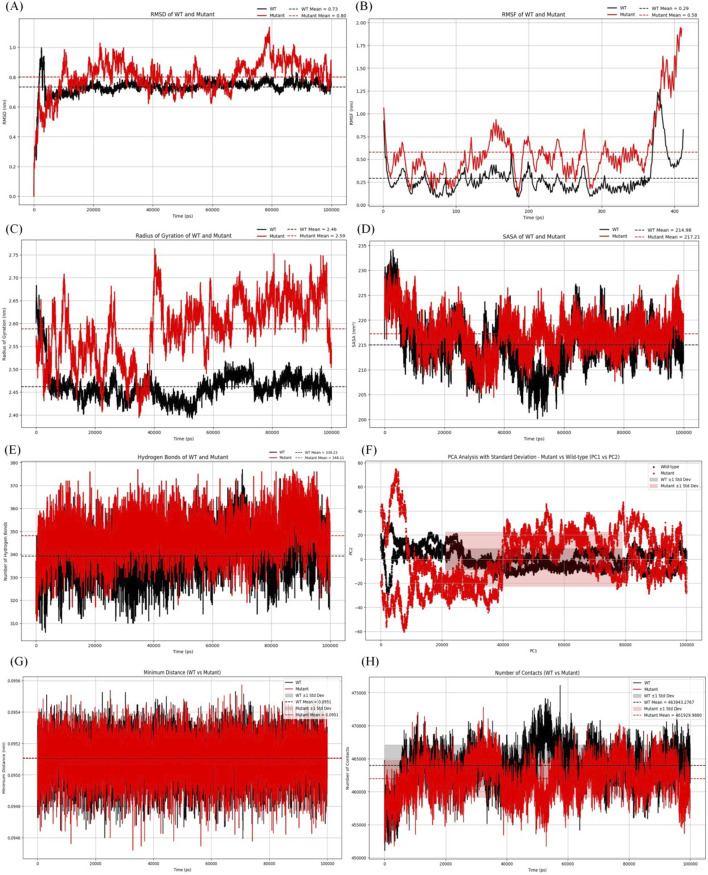
Analysis of molecular dynamics simulation results. **(A)** RMSD of the wild-type and mutant structures.**(B)** RMSF of the wild-type and mutant structures. **(C)** Rg of the wild-type and mutant structures. **(D)** SASA of the wild-type and mutant structures. **(E)** The hydrogen bonding number of the wild-type and mutant structures. **(F)** The graph for PCA of the wild-type and mutant structures. **(G)** Minimum contact distance diagram of the wild-type and mutant structures.**(H)** Contact digitogram of the wild-type and mutant structures.

As shown in Figure 4A, the average RMSD value of the wild type protein was 0.73 nm. The average RMSD value of the mutant was 0.8 nm, indicating that the mutant was less stable compared to the wild type protein. Additionally, significant fluctuations in RMSD were observed in the mutant after 9.8 ns.

Figure 4B showed the average RMSF value of the wild type protein was 0.29 nm, while the average RMSF value of the mutant was 0.58 nm. The results indicated that the mutant structure was more flexible than the wild type, and additionally, an increase was observed in the mutant at residues 375–400.

Figure 4C showed the average Rg of the mutant protein was 2.59 nm, while that of the wild type was 2.46 nm. After 14 ns, the Rg value of the mutant was slightly higher. The average Rg of the mutant being greater than that of the wild type indicated that it was less compact and more flexible.

Figure 4D showed the average SASA value of the mutant protein was 217.71 nm^2^, while that of the wild type protein was 214.98 nm^2^. The results indicated that the conformational stability of the mutant protein was slightly higher than that of the wild type, suggesting that the mutant exhibited stability from the perspective of solvation and surface area.

Figure 4E displayed the changes in the number of hydrogen bonds over time for the wild type and mutant proteins. The average number of intermolecular hydrogen bonds in the wild type protein was 339, while the mutant protein had an average of 348 hydrogen bonds. The results indicated that the mutant exhibited a similar number of hydrogen bonds compared to the wild type, suggesting comparable interactions in both protein forms.

According to the PCA graph (Figure 4F), the points for the mutant were noticeably more dispersed than those for the wild type, especially along PC1. The fluctuation range of the mutant protein (red area) was significantly greater than that of the wild type (gray area), particularly in the early to mid-stages, indicating that the conformations of the mutant were more unstable.

**FIGURE 5:**
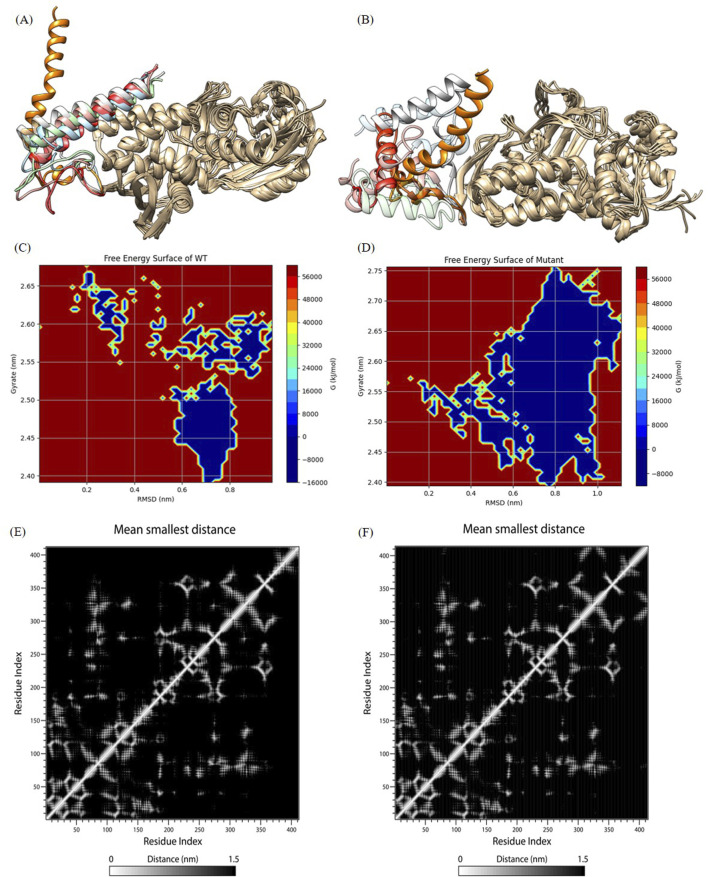
Analysis of molecular dynamics simulation results. **(A)** and **(B)** show wild-type and mutant initial structures and protein structures at 20 ns, 40 ns, 60 ns, 80 ns, 100 ns during simulation. The C-terminal helices at 20 ns, 40 ns, 60 ns, 80 ns, and 100 ns are labeled in orange, light blue, red, pink, cyan, and gray, respectively. **(C)** and **(D)** The FES analysis of the wild-type and the mutant structures. **(E)** and **(F)** Contact Matrix Diagram of the wild-type and the mutant structures.

According to the contact map analysis for the wild type and mutant (Figures 4G, H; Figure 5E, F), the wild type protein exhibited more intramolecular contacts throughout the simulation, indicating greater structural stability. In contrast, the mutant protein displayed fewer contacts during the simulation, suggesting that its structure might have been relatively loose or destabilized due to the mutation, especially after residue 360. The mutation led to a reduction in residue interactions within the protein structure, which could have affected the overall stability of the protein.

We outputed the wild-type and mutant protein structures during simulations for observing conformation changes in the loop region as well as the C-terminal helix (shown in Figures 5A, B). Consistent with the RMSF demonstration, the wild-type structure showed large conformation changes in the loop region, while the C-terminal helix only showed a shift in position and converged toward the protein core, but the C-terminal helix orientation was not changed, which was a normal phenomenon. However, both the loop region and the C-terminal helix of the mutant structure showed large structural changes, and nearly half of the C-terminal helix was deconvoluted and showed secondary structure disorder, i.e., the C-terminal helix orientation was flipped. This suggested that the mutated protein structure may not be able to hold the C-terminal helix secondary structure (we try to explain this phenomenon in the Discussion section), which was a key functional structure involved in tetrameric polymerization. We all know that protein polymers typically exhibit greater activity and more complex functions. Protein polymers could achieve more complex and efficient biological functions, such as catalytic reactions, signaling, and structural support, through interactions between multiple monomers. In addition, protein polymers were typically more stable than monomers and were better able to adapt to different environments and conditions. Therefore, the molecular dynamics simulation results indicated that the *p.Ser376-Asn378delinsArg* mutation destabilized the C-terminal helical domain, leading to a loss of oligomeric function.

As shown in Figure 5C (wild type) and Figure 5D (mutant), blue indicating lower energy and red indicating higher energy. The mutant exhibited lower energy during the simulation, while the wild type had higher energy. This suggested that the mutant experienced greater fluctuations post-mutation, but these fluctuations occurred in a lower free energy region.

## 4 Discussion

CTLN1 encompasses a spectrum of clinical phenotypes. Patients presenting with fatal neonatal hyperammonemia are classified as classical citrullinemia, while those with late-onset and/or mild symptoms are referred to as mild citrullinemia (Beaudet et al., 1986; Diez-Fernandez et al., 2017). Biophysical characterization of these patients reveals elevated levels of blood ammonia and citrulline (Enns et al., 2007; Häberle and Rubio, 2014). In this study, we described a Chinese child with classical CTLN1, who presented with severe clinical manifestations, hyperammonemia (exceeded the upper limit of the reference range by 20 times) and elevated citrulline levels (exceeded the upper limit of the reference range by 40 times), had experienced delays in motor and language development due to the delay in treatment unfortunately. Recently, Kang et al. (2021) reported a case of CTLN1 patient who received nutritional intervention for over 3 years, maintained normal development and achieved long-term survival. Moarefian et al. (2022) suggested that detection of genetic carriers in families affectedby CTLN1 would be a useful means of diagnosis to pre-vent severe forms while managing the more curableforms at birth. Typical cases are easy to diagnose; however, mild or asymptomatic patients may only exhibit a slight to moderate increase in blood citrulline levels and normal blood ammonia, making clear diagnosis and prognosis challenging. Therefore, genetic diagnosis is particularly important in these instances.

Indeed, only sporadic cases of CTLN1 have been reported in China, often identified without functional analysis, and deletion-insertion mutations have never been reported worldwide (Lin et al., 2019). Whole-exome analysis revealed a homozygous mutation consisting of a deletion-insertion variant *c.1128_1134delinsG(p.Ser376_Asn378delinsArg)* located in exon15 and a large fragment heterozygous deletion (exon13-16) in this patient. The transformation was uncommon in the population and had not been previously reported. This mutation contributed to the expanding spectrum of mutations associated with the ASS1 gene. Overall, the clinical significance of this deletion-insertion mutant was still not understandable through searching database. This mutation was an in-frame deletion-insertion mutation that did not cause a frameshift. Typically, it should not affect the protein’s structure or function and was likely benign. However, this patient had classical and severe symptoms of CTLN1. Bijarnia-Mahay et al. (2018) reported that the p.Gly390Arg mutation in the ASS1 gene was one of the most common mutations among CTLN1 patients, many of whom exhibited severe symptoms during the neonatal period, along with a high mortality rate. Given that the location of this mutation was very close to the one discussed in this article, this further supported the likelihood that the mutation was pathogenic. Additionally, Berning et al. (2008) found that patients with the p.Gly362Val gene mutation exhibited symptoms of CTLN1. Since this mutation was located at the junction between the synthase domain and the C-terminal domain, its position was similar to the mutation reported here, which further strengthened the argument for the potential pathogenicity of the mutation in our study. In conclusion, we had reason to believe that this mutation was pathogenic.

In the ASS1 protein, the C-terminal helix is a core component of the tetramer structure (Karlberg et al., 2008). The center of the tetramer creates a cavity that allows solvent to pass through, with the inner surface of this cavity containing the synthase active sites of the ASS1 protein. This cavity significantly enhances the synergistic interactions between the active sites, improving catalytic efficiency. Compared to monomers or dimers, the tetrameric structure is more stable, creating a “shell” that protects the active cavity, making it more resilient to changes in the external environment. This structure not only prevents the denaturation and degradation of the active sites but also ensures the maintenance of ASS1 protein function under adverse conditions. Liu et al. (2023) revealed the c.1048C>T variant in ASS1 causes a loss of 63 amino acids, including the entire C-terminal oligomerization domain and validated the pathogenicity using *in silico* protein modeling. In this study, the analysis of experimental data and the structures derived from the simulation showed that the mutant protein structure cannot maintain the secondary structure of the C-terminal helix, which predicted that the mutated protein would most likely fail to form oligomeric proteins, which would further affect the function or activity of ASS1 protein.

In clinical practice, plasma amino acid analyses should be conducted promptly for patients suspected of having CTLN1. Additionally, genetic analysis is essential for accurate diagnosis, early treatment, and improving pediatric survival rates, while also providing a reliable foundation for future prenatal diagnosis and genetic counseling. The *in silico analysis* of mutations that aligned with clinical manifestations of the disease but did not cause a frameshift clarified the pathogenicity of the mutations, laying the groundwork for future intervention targets. Our approach and findings offer new insights for explaining the discrepancies between the clinical results and genetic outcomes. However, the relationship between genotype and phenotype requires further exploration through biochemical experiments, and additional biological experimental methods are needed to validate these observations.

## Data Availability

The data presented in the study are deposited in the GSA-human repository (https://ngdc.cncb.ac.cn/gsa-human/), accession number HRA005194.
